# Antipredator behaviour as a major determinant of prey altitudinal movements: the wolf and the chamois

**DOI:** 10.1186/s12983-025-00559-1

**Published:** 2025-08-21

**Authors:** Valerio Orazi, Matteo Panaccio, Sandro Lovari, Irene Belardi, Achaz von Hardenberg, Bruno Bassano, Francesco Ferretti

**Affiliations:** 1 Department of Life Sciences, University of Siena, Via P.A. Mattioli 4, 53100 Siena, Italy; 2 Department of Biological Sciences, University of Chester, Parkgate Road, Chester, CH1 4BJ UK; 3 Maremma Natural History Museum, Strada Corsini 5, 58100 Grosseto, Italy; 4 Department of Earth and Environmental Sciences, University of Pavia, 27000 Pavia, Italy; 5 Alpine Wildlife Research Centre, Gran Paradiso National Park, 10080 Noasca, Italy; 6 NBFC, National Biodiversity Future Center, 90133 Palermo, Italy

**Keywords:** Predator–prey, Large carnivores, Mountain ungulates, Antipredator behaviour

## Abstract

**Background:**

Predators have the potential to affect prey ecology through both direct effects on population dynamics or indirect effects on behaviour, e.g., by triggering antipredator strategies. Direct effects of predation on single prey species may be limited in ecosystems hosting alternative prey, possibly being overwhelmed by indirect effects. The novel exposure to a predator would provide the opportunity to test for immediate prey responses, but information is scanty for areas recolonised by carnivores. We took advantage of the natural expansion of the wolf *Canis lupus* in a protected area of western Alps hosting five ungulate species to test the potential for direct versus indirect effects on the main prey, i.e., a widespread mountain herbivore (the Northern chamois *Rupicapra rupicapra*). After verifying the contribution of the latter to the diet of the former, we used a semi-experimental (before vs. after) approach by comparing chamois demography, elevation used and group size between two valleys with different recolonisation time (Site A: medium-term vs. Site B: short-term).

**Results:**

Scat analyses (*N* = 335 samples) indicated that chamois were the staple in the wolf diet in both valleys. Analyses of counts throughout 21 years supported no direct effect of wolf on chamois abundance and survival. Following wolf recolonisation, female chamois (*n* = 3594 observations) in Site A were observed at average elevations 137 m higher compared to the former period, and a concurrent decrease of group size was reported; these effects were not detected in Site B. The same trend was not observed in temperature, precipitation or NDVI, providing no support to a weather- or resource-mediated uplift.

**Conclusions:**

Although direct/indirect effects of current changes in weather patterns on the observed uplift of chamois may not be ruled out, our results suggest antipredator behaviour as a main determinant of chamois upshift. Finally, we discuss the role of indirect versus direct short-term prey responses in complex ecosystems.

**Supplementary Information:**

The online version contains supplementary material available at 10.1186/s12983-025-00559-1.

## Introduction

Although herbivore behaviour and ecology are largely affected by resource availability and distribution, as well as social factors [1], there is increasing interest in identifying the role of top-down processes [2–4]. Predation risk is a powerful driver influencing interspecific interactions [5], through direct (i.e., lethal) or indirect (i.e., nonlethal) effects on prey. As a direct consequence of predation, prey abundance and demography may be affected [6–9]. This is expected to occur especially when predators select consistently for a single species [10–12]. In healthy multi-prey ecosystems, predation pressure may be distributed across several species, thus decreasing the impact of predators on each of them [13], unless a strong preference occurs for a particular species [14, 15].

Direct effects of predation could be exceeded by indirect, non-consumptive ones [16], involving behavioural changes in prey individuals triggered by predation risk [17, 18]. Antipredator responses may include increased vigilance [19, 20], spatial and/or temporal avoidance [21–24], group size variations [25–27], or increased selection for safer habitats [28–30]. Under some conditions, effects of predation may propagate across trophic webs [31–33]. However, the nature and strength of antipredator responses are not ubiquitous, being strongly influenced by species-specific and ecological factors [34]. Indirect effects triggered by the risk of predation may bear costs for individual fitness, e.g. when an antipredator response involves an increased selection for sub-optimal habitats, increased stress levels or reduced food intake [33–35]. If this process occurs, indirect and direct effects may become additive: in other terms, prey demography may be affected by a combination of direct predation and demographic costs of antipredator behavior [36].

Understanding consumptive and non-consumptive effects of top carnivores on prey is a major challenge in predator–prey studies, particularly in areas recently recolonised by predators [37]. Prey populations facing unfamiliar predators may be exposed to a higher predation mortality [38, 39] and develop a variety of direct and indirect responses. For example, wapiti *Cervus canadensis* have been found to cope with predation of recolonising wolves *Canis lupus* by shifting to structurally complex refuge areas [40]. In predator-free areas of North America, moose *Alces alces* have been found to be less responsive to predator acoustic cues compared to moose living in sites with historical predator occurrence [39], suggesting a higher vulnerability to unfamiliar predators.

The nature and strength of adaptive responses to predation risk should be influenced by the intensity of predation pressure [23, 41]. Predation pressure on different prey species may not be fixed through time as carnivore food habits may show variations through the years, possibly in response to changes in prey abundance or behaviour [42–44]. Data on predator food habits are therefore necessary to formulate appropriate hypotheses on prey-predator relationships. However, data linking prey responses to predator food habits are scarce, especially for European ecosystems.

Group size variation in response to predation risk has been reported for many species [26, 27, 45, 46]. The advantages of being in large groups reside in the dilution and detection effects, as well as in group defence [47]. Group living seems to have particular advantages in open habitats [48, 49]. Moving to safer habitats as an immediate antipredator response has been reported for species living in low human density areas [50–52]. On mountainous grounds, moving to safer habitats may imply an increased use of sites located at higher elevations, close to rocky areas. Mountain ungulates are well adapted to cope with steep and precipitous terrain, and tend to seek refuge on cliffs and rugged terrain [53–56]. In European countries, where the wolf is the most widespread top predator [57], mountain ungulates are often a secondary prey, although locally they can become the carnivore’s staple food [58]. Species living in areas with predominantly open habitats such as alpine meadows would be expected to increase group size to reduce predation risk [59]. As a consequence of predation pressure, mountain ungulates would be also expected to increasingly use safer habitats at higher elevations or closer to rocky areas. However, information on responses of mountain herbivores to recolonising predators is still scarce [9, 34, 60]. While population growth rate and fawn body mass of roe deer *Capreolus capreolus* have been negatively affected by wolf *Canis lupus* recolonisation in the French pre-Alps [61], the effects of wolf recolonisation on populations of mountain-dwelling prey species have not been clarified (for chamois: [62]).

We aimed to fill this gap and considered the responses of the most widespread mountain ungulate in Europe, i.e., the Northern chamois *Rupicapra rupicapra,* to recolonising wolves. We worked in a multi-prey ecosystem including also the Alpine ibex *Capra ibex*, the roe deer, the red deer *Cervus elaphus*, and the wild boar *Sus scrofa*. In Alpine areas, the chamois is a substantial prey to wolves, although predation is usually distributed across several prey species [63, 64]. Thus, we could expect a low potential for direct effects of predation on a single species, considering that the wolf can exploit several alternative prey. On the other hand, main prey may have a greater potential for developing antipredator behavioural responses.

The spatiotemporal variation commonly occurring in colonisation processes provides the opportunity to compare prey responses across sites with different colonisation times. We have compared chamois responses across sectors with different wolf recolonisation history, by assessing (1) the chamois importance in the wolf diet; (2) the direct effects of the wolf return on chamois population dynamics; (3) the indirect effects on group size and spatial patterns of female chamois groups. We expected that, after the wolf returned to the area, (1) limited variation in chamois abundance, survival and/or birth rates would occur, and (2) an increase should be expected in group size and elevation used by chamois groups of females with offspring.

## Materials and methods

### Study area

This study was conducted in two adjacent valleys of the Western Italian Alps, in the Gran Paradiso National Park (Orco, 220 km^2^; and Soana, 116 km^2^, valleys) with a total surface of 336 km^2^. These valleys are included in the Piedmont sector of the park. Hunting has been strictly forbidden since the 1920s, with the exception of a few selective (older males) trophy hunts authorised until the 1960s; culling of wild boar has been conducted by park rangers since the 1990s. The environment is typically alpine, with an elevational range of 800–4061 m; at higher elevations (1400–2000 m) forests are mainly represented by conifers (dominated by larch, *Larix decidua*), while mixed broad-leaved forests (ash, hazelnut, chestnut) are widespread at lower elevations. Above the tree line, there are alpine grasslands, replaced by rocky areas, glaciers and screes above 2900 m, with low to absent vegetation cover. The climate is typical of the alpine regions: cold winters (average temperature: *c*. 2 °C, average minimum temperature: *c.* − 10.5 °C, average maximum temperature: *c.* 9.5 °C) with abundant snow and short, cool summers (average temperature: *c.* 20 °C, average minimum temperature: *c*. 9 °C, average maximum temperature: *c.* 28 °C). A fairly rich mammal community is present: apart from the Northern chamois, other ungulates are the ibex, the roe deer, the wild boar and the red deer; the last two species are present at low densities, mainly below the tree line (c. 2500 m asl). The mesocarnivore community is composed of red fox *Vulpes vulpes*, badger *Meles meles*, stone marten *Martes foina*, pine marten *Martes martes* and stoat *Mustela erminea*. Other meso-mammals are the marmot *Marmota marmota*, European brown hare *Lepus europaeus* and Alpine hare *Lepus timidus*. The wolf is the only large carnivore permanently present in the park, where it returned in the Aosta Valley sector in 2007 after about a century of absence [64, 65]. Wolves had a different colonisation history in the valleys included in our study area. An ongoing monitoring programme has revealed that the wolf was virtually absent or rare in the Piedmont sector of the park, in 2011 [66]. In Soana valley (our Site A), a pair formed in 2012 and the first (assessed) reproduction occurred in 2013, whereas in Orco valley (our Site B) reproduction was first reported in 2017 (Gran Paradiso National Park, internal report, 2018).

### Data collection and analyses

#### Wolf food habits

335 wolf scats were collected by park rangers both opportunistically and along research paths (total length: 94 km, walked monthly) from October 2019 to September 2022 (Site A: 221; Site B: 114). Wolf scats were identified according to size, shape, content, location and smell [67, 68]. No other large predator was present and free-roaming dogs were near-absent. Dubious scats were discarded from analyses. All scats were geo-referenced, marked with an individual ID code and then stored in a freezer. All samples were put in an oven at 80 °C for > 3 h to inactivate parasites; after that, the scats were washed through a 1–3 mm sieve to separate undigested material such as hair and bones [9]. We used macro- and microscopical evaluations by following atlases [69, 70] and by comparing the samples with a reference collection of hair collected from species in our study area, to correctly identify prey hair in scats. This analysis was conducted by a trained operator (IB), after completion of a blind test with at least 95% of correct identifications on 75 samples. Three metrics were used for each food item [67]: absolute frequency (percentage of scats including that category), relative frequency (number of scats containing that category, divided by the number of vertebrate food items and expressed as a percentage) and estimated volume. The volume was estimated visually, referred to the median value of the relative volumetric class: 1–5, 6–25, 26–50, 51–75, 76–95 and > 95% and expressed as follows: (total estimated volume of each prey species/ total estimated volume of all prey species) × 100 [71]. In order to build 95% confidence intervals, each of the three indices was bootstrapped with 1000 resamplings.

#### Chamois data collection

The Park Agency conducts annual counts to monitor chamois numbers. Chamois block counts were performed by experienced, trained park rangers: visual counts were simultaneously performed along the same routes and from the same vantage points every year, always in two days within the first week of September [72]. In case of bad weather, the survey was delayed to the first available date. For each chamois sighting, the following data were recorded: number of individuals in the group, sex and age classes, as well as its location within a 250 × 250 m georeferenced grid. In particular, the geographic coordinates of the centre of each grid cell where chamois were located were assigned to each sighting.

#### Direct effects: population dynamics

We investigated the potential for a direct effect of the wolf on chamois population dynamics using chamois count data from 2000 to 2020. We considered the year of first wolf reproduction in each valley as a sign of re-establishment in that area (Site A - Soana: 2013; Site B - Orco: 2017) and its effects on several parameters describing chamois population dynamics. In particular, we considered both the total chamois population (i.e., total number of counted chamois), and each sex/age class separately. We analysed the following parameters: (1) population growth rate, (2) kid survival, and (3) kid:female ratio. Growth rate was estimated for the whole population and separately for each sex, as the slope of a Generalized Linear Model built on chamois count data for the entire target period. The number of counted chamois was fitted as the response variable, modelled by using a Poisson distribution. Year was considered as the predictor. Kid survival was estimated for each year *t* as the number of yearlings in year *t* + 1 divided by the number of kids in year *t*. Kids-to-female ratio was estimated in each year as the number of kids counted in a year divided by the number of adult females.

We tested whether all the population parameters of chamois differed between years after versus before wolf settlement. For the Orco valley (Site B), the small number of years (n = 3) with wolf presence prevented us from conducting robust analyses. Thus, we focused mainly on Soana valley (Site A), where the wolf started reproducing five years earlier, for a total of eight years. We compared growth rates, kid survival and kid:female ratio after versus before 2013 in Soana. We made the same comparison (after vs*.* before 2013) also for Orco valley. In this way, we tested whether chamois population dynamics in Soana valley was different compared to an ecologically similar Park valley where wolf presence was absent or intermittent [73], i.e., whether potential changes in demographic parameters in Soana were associated with wolf settlement or occurred consistently. To compare population parameters in Soana, we used two approaches. First, we compared the growth rates of each sex, kid survival and kid:female ratio, before and after 2013, between Soana and Orco valleys through *t*-tests. Second, we included the wolf presence in Soana valley (yes or no) as a predictor in the same population Generalized Linear Model used to estimate the population growth rates.

#### Indirect effects: elevation and group size

As a rationale for our analyses, we considered the “antipredator response” hypothesis more supported if (a) changes in chamois elevation and/or group size would occur in Soana valley (Site A) after 2013, but not in the Orco valley (Site B) during the same period, and (b) no concurrent variations in temperature and NDVI would occur between the two periods. For this purpose, we used a semi-experimental BACI (Before-After Control-Impact) design.

First, for Site A (Soana) and Site B (Orco) separately, we analysed inter-annual variations of (1) chamois elevation, i.e., the mean elevation across all the sightings in each year, (2) chamois mean group size across all the sightings in each year, (3) temperature and precipitation trends (see below), (4) NDVI, and tested for both the occurrence of statistically significant trends and whether these trends differed between periods before versus after wolf settlement. As a second step, we modelled whether the elevation and the group size of chamois sightings in each valley differed between years with wolf absent and present, accounting for environmental and weather predictors (see below).

Temperature and precipitation data were downloaded from two different weather stations, one for each valley: Serrù station for Orco valley and Piamprato station for Soana valley. Both stations are located within the chamois altitudinal range (2300 m a.s.l. and 1600 m a.s.l. respectively) and thus considered to be representative of the conditions experienced by animals. We used several weather metrics: average summer maximum temperature, annual maximum temperature, maximum temperatures during the count days, total summer precipitation and total precipitation in August (i.e., the month preceding counts). In R 4.1.3 (package “terra”, [74]) we generated the centroids of each cell of the georeferenced grid that was used to record the observations, and derived elevation and slope rasters using a Digital Terrain Model (20 m resolution, then aggregated to 250 m to match the survey grid resolution) provided by the Gran Paradiso National Park Agency; we used the same raster to calculate the Terrain Ruggedness Index (TRI, [75]) relative to the centroid of each cell with a recorded observation. Weekly NDVI values from 2000 to 2020 were obtained from the MODIS TERRA satellites (250 m resolution, link in Additional file). For each year, we extracted the average cell values of the week between the 241st and the 248th day of the year, to center the fixed survey period, using the ‘terra’ R package. We used the same package to calculate the mean values of the cell of each observation and the ones of its adjacent cells. The mean NDVI trend was also calculated for each 200-m zone, starting from 1800 m (forest edge), to check whether meadows at different elevation classes showed the same inter-annual pattern of vegetation productivity, which may be linked to chamois movements. The package “trend” [76] was used to perform the non-parametric Pettitt’s test [77] for change-point detection in the trend analysis and to interpret correctly the time series of the different metrics. When a change point (K) is detected, the test provides an estimate of when it occurs and if the two samples defined by it (i.e., before vs. after) come from the same distribution. With this approach, we checked whether a change point in the series occurred in the elevation of chamois sightings and in chamois group size and, if so, if it was concomitant with the year of wolf first reproduction in Soana (Site A: 2013). To evaluate whether environmental variables potentially driving chamois altitudinal movements also showed the same inter-annual variation, we applied the Pettitt’s test also to trend analyses of temperature and global NDVI in each valley. A detection of a trend in chamois elevation and/or group size but not in temperature and/or NDVI would support us to consider these environmental variables as not being major determinants of chamois responses.

For the second phase, to model the differences between periods and valleys we considered the 2013 as a threshold and categorised years as occurring “after” versus “before” that date. A change of chamois elevation and/or group size after 2013 only in Site A (Soana) but not in Site B (Orco) would provide support to a role of the wolf in triggering it. Using Generalized Linear Mixed Models, we assessed whether female group size and elevation of female groups were different between valleys, as well as before and after wolf recolonisation, accounting for NDVI, temperature, precipitation, and topographic variables (slope and TRI). To avoid confounding effects of interannual variability in the number of kids and yearlings, we considered the number of adults only. Before modeling, a correlation matrix was calculated (see Additional file 1, S2) to explore possible multicollinearity in the set of covariates: r values > 0.75 were considered to be too high [78]. Among the topographic variables, slope was highly correlated with TRI: we therefore discarded TRI as considered to be less informative than slope, being an index rather than a measure. Maximum temperature during the survey was correlated with average maximum temperature during summer. Therefore, we fitted two global models with alternatively the first or the second of these variables, checking for the lowest AIC score between them to select the best one. After this step, among the weather variables, we retained only the maximum temperature during the survey period, plus the total precipitation during summer and the total precipitation during August. We fitted the mixed models using the package “glmmTMB” [79]; group size and elevation of each observation were the response variables, and the year was set as a random factor in both cases. Differences between pre and post-colonisation were tested by inserting an interaction between wolf presence in Soana valley in each year and the valley (Soana or Orco). We set a Poisson distribution for group size and a Gaussian one for elevation. Model selection was based on lower-AICc and nesting rule criteria (selecting models with a difference of AICc < 2 and with equal or less parameters than the best fitting model, [80]), using the package “MuMIn”. Model performance was assessed with the package “DHARMa” [81] and “performance” [82].

## Results

### Food habits

**Fig. 1:**
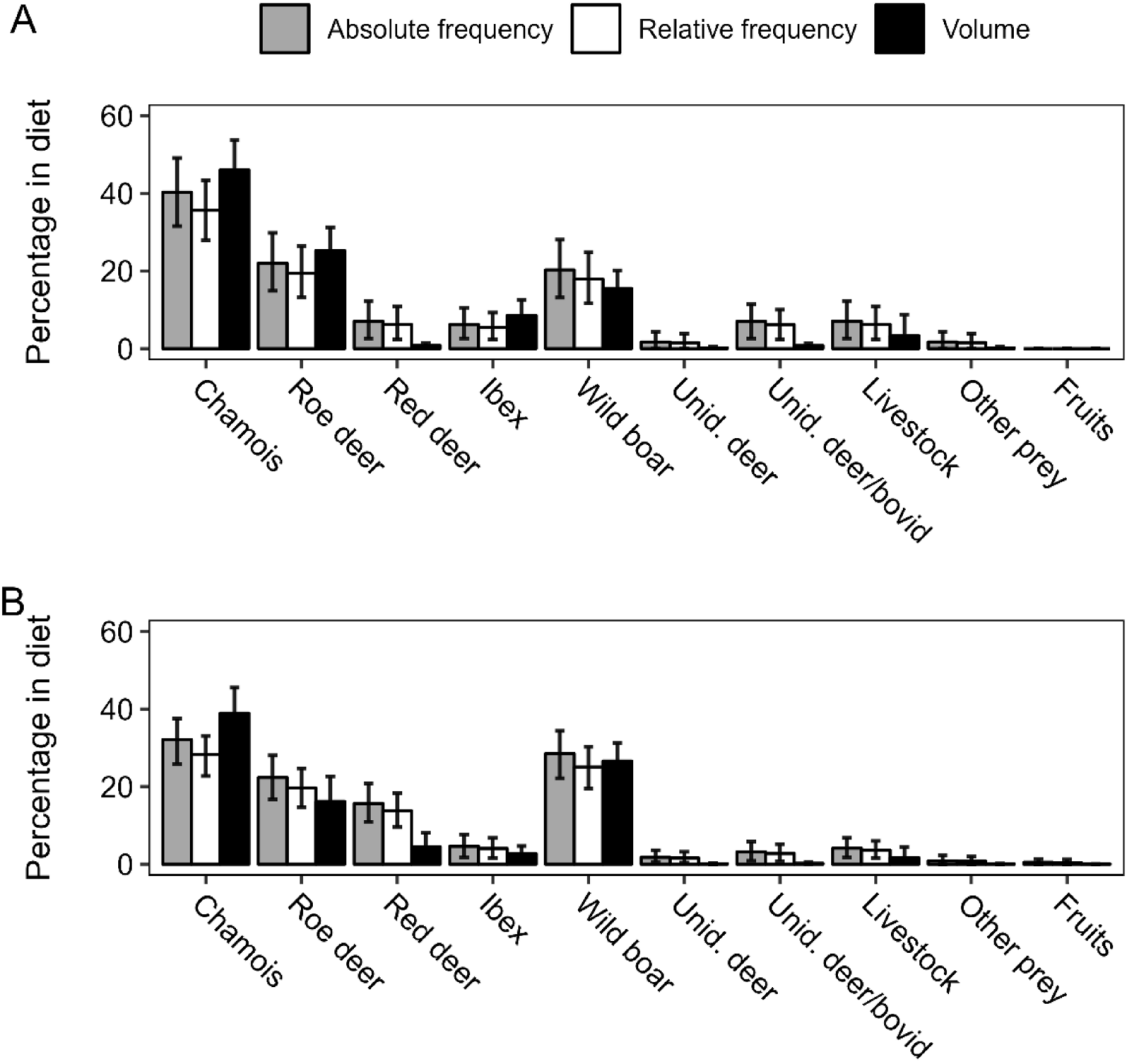
Absolute frequency, relative frequency, estimated volume and their relative bootstrapped 95% confidence intervals (1000 resamplings) of each food category representing the wolf diet in Orco (**A**) and Soana (**B**), during the period October 2019–September 2022

Chamois accounted for more than 31% of the wolf diet in all the calculated metrics in both valleys, resulting in being the main prey in our study area. The roe deer and the wild boar were the second prey in Orco and in Soana, respectively (Fig. 1). Red deer showed *c.* 15% occurrence in the wolf diet in Soana, while less than 10% in Orco. Other ungulates were far less used (i.e., < 7%), and other prey occurred only occasionally (Fig. 1).

### Chamois population dynamics

**Fig. 2:**
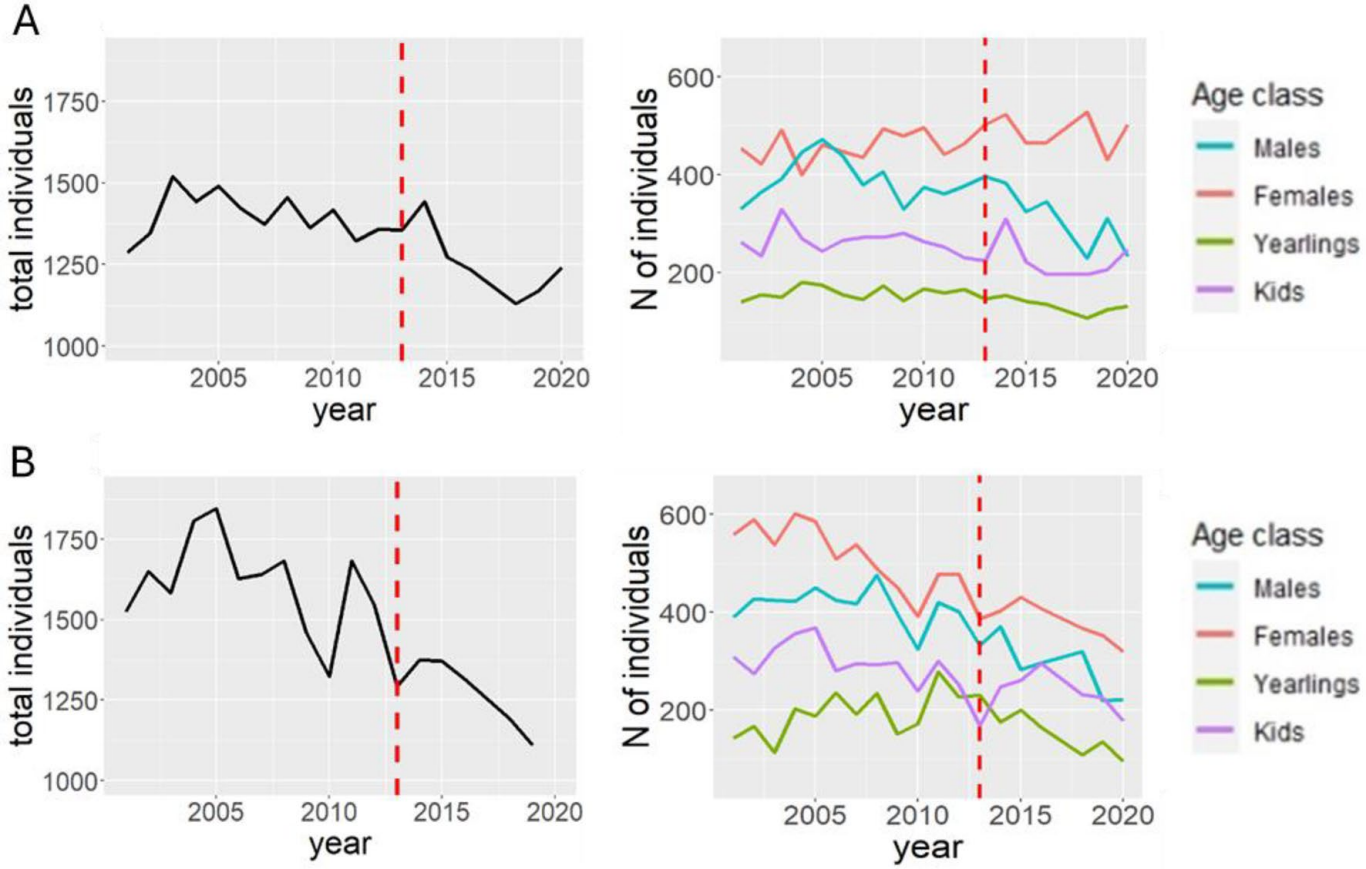
Population dynamics of chamois in Soana valley (**A**) and Orco valley (**B**). Total population is represented on the left, while population divided by age class is on the right. The dashed line represents the year 2013, corresponding to the stable wolf presence in Soana valley

Dynamics of our study population between 2000 and 2020, in Soana and Orco valleys and in the rest of the park territory, are set out in Fig. 2, while population parameters are shown in Table 1.

**Table 1:** Population parameters of chamois

Period	Total pop λ	Males λ	Females λ	Kid survival	Kid:Female ratio
*Soana valley*					
Before 2013	0.998 ± 0.002	0.996 ± 0.004	1.006 ± 0.004	0.60 ± 0.05	0.58 ± 0.05
After 2013	0.976 ± 0.004***	0.930 ± 0.009***	0.994 ± 0.007 ns	0.60 ± 0.07 ns	0.47 ± 0.07***
*Orco valley*					
Before 2013	0.992 ± 0.002	0.994 ± 0.004	0.973 ± 0.004	0.65 ± 0.19	0.58 ± 0.05
After 2013	0.958 ± 0.004***	0.941 ± 0.009***	0.971 ± 0.008 ns	0.68 ± 0.25 ns	0.60 ± 0.09 ns

λ = population growth rate. Asterisks indicate the significance of statistical differences between years before and after 2013. ***p < 0.001; ns: p > 0.05

For both the valleys, the GLM supported an interactive effect of year and period on chamois total abundance, with a decrease of total population size after 2013 (i.e., before vs. after 2013; Soana: *β* = − 0.022 ± 0.005; Orco: *β* = − 0.034 ± 0.005). The same result was obtained for the male portion of the chamois population (Soana: *β* = − 0.065 ± 0.009; Orco: β = − 0.053 ± 0.01). There was no support to a decrease of female numbers after 2013.

Looking at growth rates, a decrease was detected after 2013 in the valleys for the total population and for males, whereas for females this result was not supported (Table 1). Kid survival did not differ before and after 2013 in either valleys, while kid:female ratio was lower after wolf recolonisation only in Soana valley (Table 1).

When considering only the period after 2013, the growth rate of the total population was greater in Soana than in Orco (t = 3.18, p = 0.001). Male growth rates were comparable between Soana and Orco (t = 0.86, p = 0.38), while female growth rates were greater in Soana than in Orco (t = 2.16, p = 0.03).

### Elevation and group size

**Fig. 3:**
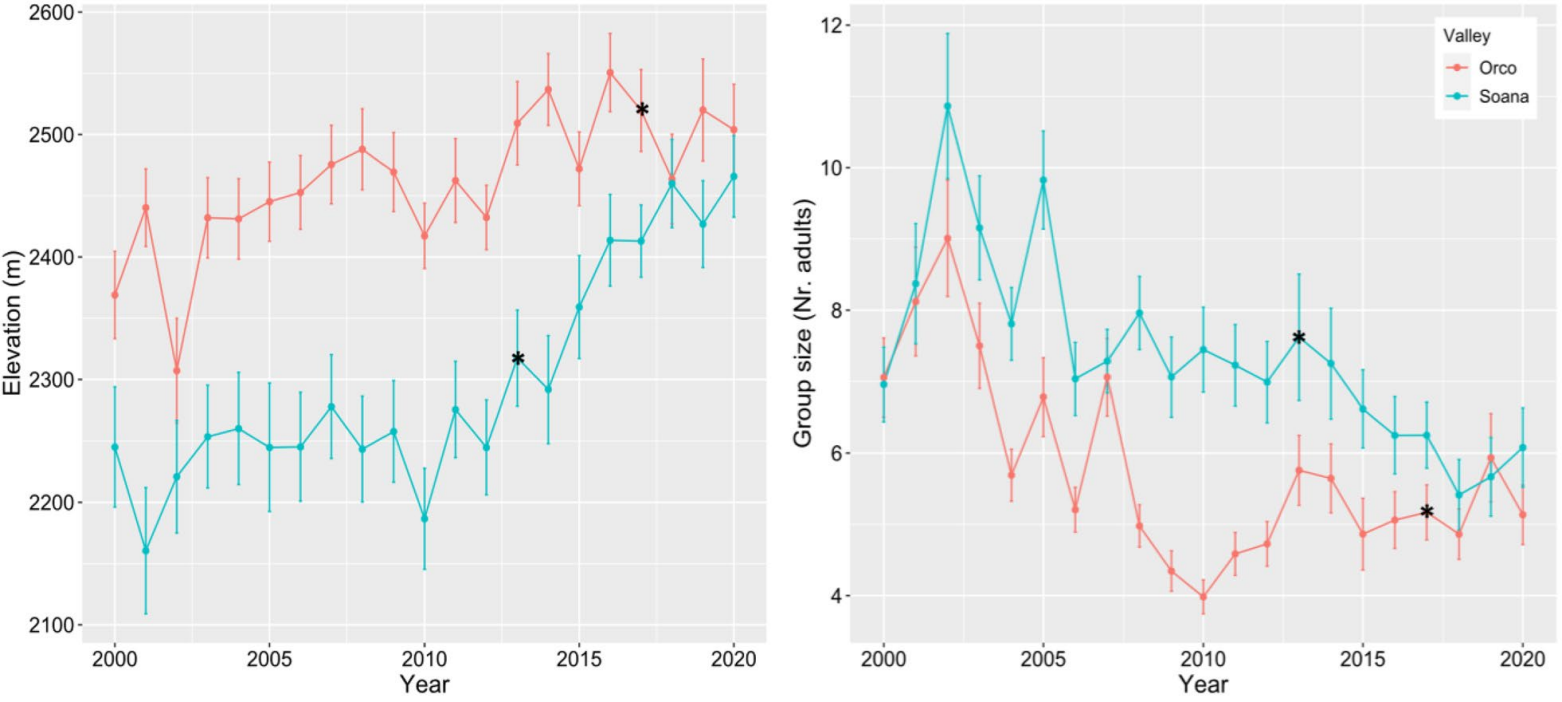
Mean and standard error of elevation (left) and group size (right) trends in Orco and Soana valleys. Black stars mark the year of the first assessed wolf reproduction in each valley

A total number of 3548 female group observations were included in this analysis. The mean elevation of chamois groups was relatively stable before wolf reproduction in Soana (Fig. 3), but it showed a steep increase after 2013 (K = 2012, p-val = 0.002, U* = 104). In Orco, chamois groups were observed at higher altitudes than Soana before wolf reproduction; a slight and not significant increase was observed after 2013 (Pettitt’s test, K = 2013, p-val = 0.08, U* = 94).

The mean group size showed a decrease in Soana valley over the whole 20-year period (Fig. 3). This decrease was significantly greater immediately after wolf colonisation (Pettitt’s test, probable change point K = 2014, p-val = 0.01, U* = 90). This trend was different in Orco valley (Fig. 3), were group size decreased until 2010 and then slightly increased again (K = 2008, p-val = 0.008, U* = 94).

**Fig. 4:**
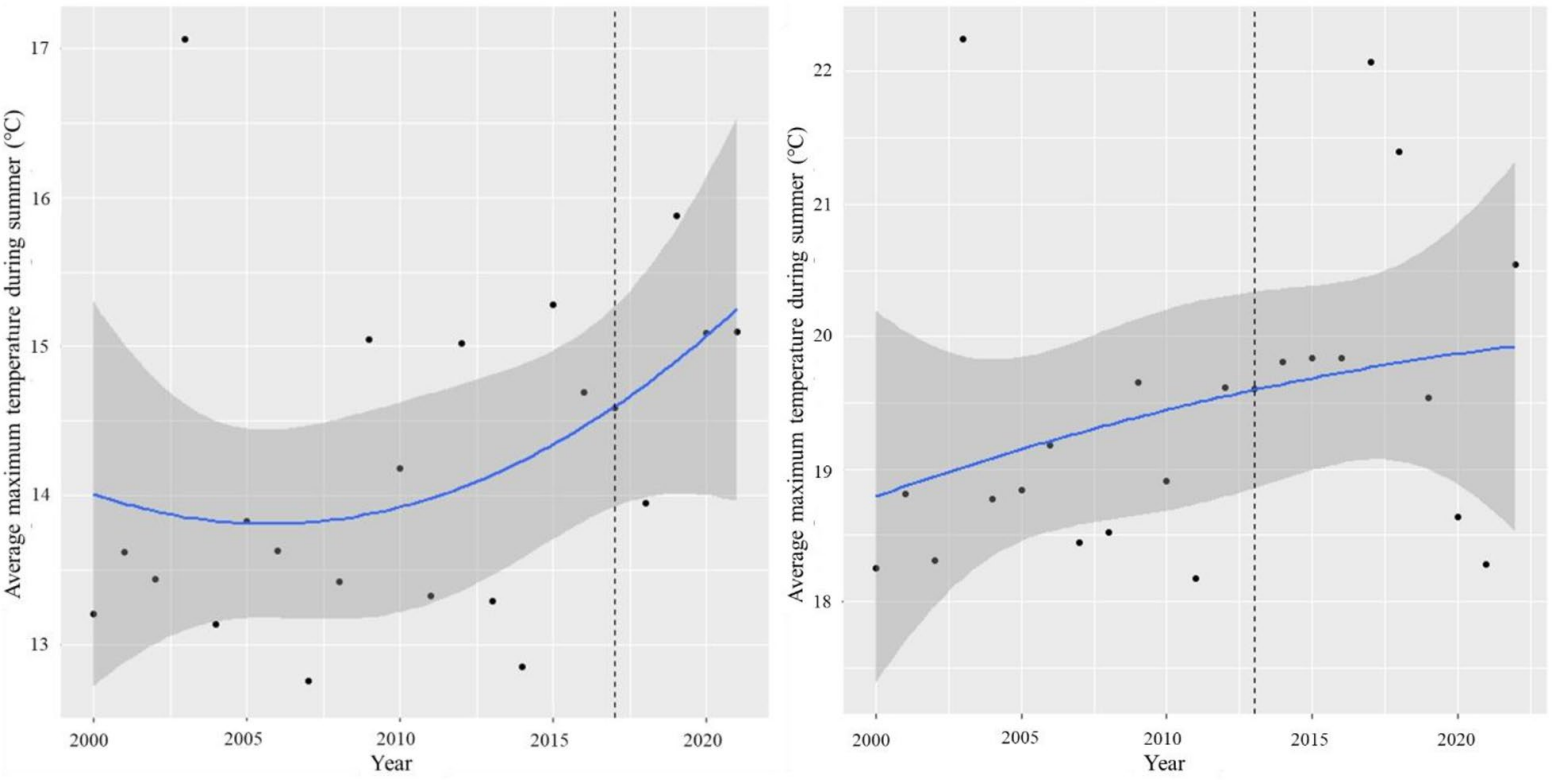
Average maximum temperature trend during summer in Orco (left) and Soana (right) valleys from 2000 to 2021, and relative confidence intervals (grey). Vertical dotted lines represent the reference year of wolf colonization in each valley

As to temperature trends, irregular fluctuations were recorded in both valleys for maximum temperatures during the survey period while a slow, quasi-linear increase was recorded for average maximum temperatures in summer (Fig. 4). No change point was detected along both these time series in either Soana (respectively: K = 2003, U* = 49, p-val = 0.45 and K = 2011, U* = 70, p-val = 0.10) or Orco (respectively: K = 2003, U* = 45, p-val = 0.57 and K = 2014, U* = 62, p-val = 0.18). A similar result was observed for total precipitations during August and during the summer months, in both Soana (K = 2, U* = 28, p-val = 1 and K = 10, U* = 43, p-val = 0.63) and Orco (K = 16, U* = 40, p-val = 0.74 and K = 6, U* = 34, p-val = 0.97). Zonal NDVI values (Additional file 1, S3) showed inter-annual fluctuations at lower to intermediate elevations within the survey week, but no significant overall trend was observed in any of the two valleys (Soana: K = 15, U* = 64, p-val = 0.15. Orco: K = 15, U* = 42, p-value = 0.6718).

**Fig. 5:**
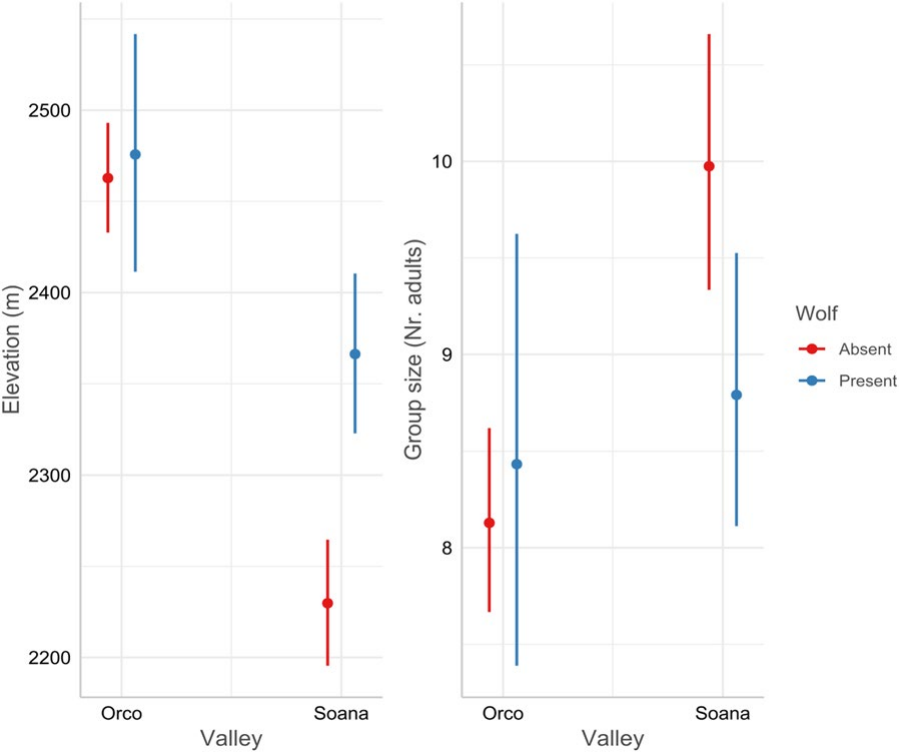
Elevation (left) and group size (right) of female chamois in each valley comparing before and after wolf recolonisation, as predicted by the best selected model

For the elevation of female groups, the set of models with a difference in AIC < 2 compared to the best one are shown in Table 2. All models within this range retained the interaction between wolf and valley. The best selected model (i.e. with lower AIC and lower number of parameters, Table 3) revealed a significant increase of about 137 m in Soana valley after wolf colonisation, but not in Orco valley (Fig. 5). This model included the maximum temperature during the survey, although the effect of this variable was not supported (Table 3).

**Table 2:** Model selection table for elevation and group size of female chamois groups

Variable	Rank	Model structure	df	logLik	AIC	ΔAIC	Weight
*Elevation*	1st	Wolf + Valley + Wolf x Valley + slope + Tmax survey	**8**	− **25,674**	**51,364.6**	**0.00**	**0.343**
	2nd	Wolf + Valley + Wolf x Valley + slope	**7**	− **25,675**	**51,365.0**	**0.42**	**0.285**
	3rd	Wolf + Valley + Wolf x Valley + Tmax survey + tot.prec.august + slope	9	− 25,674	51,366.4	1.81	0.134
	4th	Wolf + Valley + Wolf x Valley + slope + tot.prec. summer	9	− 25,674	51,366.5	1.99	0.130
	5th	Wolf + Valley + Wolf x Valley + tot. prec. august + slope	8	− 25,675	51,367.0	2.39	0.108
*Group size*	1st	Wolf + Valley + Wolf x Valley + NDVI	**7**	− **10,982.87**	**21,979.8**	**0.00**	**0.248**
	2nd	Wolf + Valley + Wolf x Valley + NDVI + tot. prec. august	8	− 10,982.03	21,980.1	0.33	0.207
	3rd	Wolf + Valley + Wolf x Valley + NDVI + Tmax survey	8	− 10,982.03	21,980.1	0.34	0.207
	4th	Wolf + Valley + Wolf x Valley + NDVI + Tmax survey + tot.prec.august	9	− 10,981.13	21,980.3	0.54	0.187
	5th	Wolf + Valley + Wolf x Valley + NDVI + Tmax survey + tot.prec.summer	9	− 10,981.35	21,980.7	0.97	0.151

All models up to the fifth are presented (see Additional file 1, S6 for a complete list)

Avg.summer.Tmax, average maximum temperature during summer; Tot.prec.august, total precipitation during august; Tot.prec.summer, total precipitation during summer; Tmax survey, maximum temperature during the survey period (first week of September).). Selected models are shown in bold

**Table 3:** Variables explaining elevation and group size of chamois female groups, as estimated by the best mixed model

Response variable	Model		Covariates	*B*	*SE*	p-value
*Elevation*	Best		Wolf [Present]	0.005	0.014	0.713
			Valley [Soana]	− 0.099	0.011	< 0.01
			Slope	− 0.002	0.0002	< 0.01
			Tmax during survey	0.001	0.001	0.209
			Wolf [Present] × Valley [Soana]	0.54	0.132	< 0.01
*Group size*	Best		Wolf [Present]	0.036	0.070	0.605
			Valley [Soana]	0.204	0.028	< 0.01
			NDVI	− 0.308	0.060	< 0.01
			Wolf [Present] × Valley [Soana]	− 0.163	0.069	0.018

Regarding female group size, all models again retained the interaction between wolf and valley (Table 2 and Additional file 1, S4). The best model predicted a decrease from an average group size of 10 individuals before the wolf colonisation to 8.7 after it in Soana valley, while in Orco valley no change was supported (Fig. 5) with no effect of climatic variables but a significant effect of NDVI (Table 3).

## Discussion

Among the limiting factors which regulate populations of mountain ungulates, winter rigours are a particularly important determinant of population dynamics [83–86]. Conversely, direct effects of predation may be difficult to assess (i.e. difficulty of discriminating predation-induced mortality from emigration and other causes of death, [85]) and indirect effects on spatial behaviour of prey are also challenging. Thus, the recolonisation of areas with long-time missing predators can prove useful to get insights on predator–prey spatial dynamics.

We used the Alpine chamois as a mountain ungulate model species to simultaneously assess its importance in the wolf diet and its direct and indirect responses to wolf recolonisation. To our knowledge, this is the first study investigating the effects of the wolf on both population dynamics and spatial patterns of a mountain ungulate in the Alpine context. Although estimates derived from visual counts could underestimate population size of mountain ungulates [87], they also seem to correlate well with other reliable methods [88], even in our study area [72], and have therefore been used to estimate population trends [88–90]. Therefore, as the aim of this study was to compare population dynamics before and after wolf recolonisation rather than comparing exact population estimates, we consider our data to be adequate for this purpose. The chamois is the most widespread mountain ungulate across the Alps and other Eurasian mountain areas. Its overall population trend is stable to increasing, but several threats have been reported, e.g. human disturbance, meteorological changes, hunting and predation [91]. The quick recolonisation of the Alpine arch by the wolf [92], particularly in Italy [93], can expose chamois populations to a locally increased predation pressure to which they are no longer used, after living for *c*. one century without large terrestrial predators. In our study area, the chamois is the main prey of the wolf, followed by the roe deer, and is expected to have developed adaptive antipredator strategies [53]. Presumably, since well over 10,000 years ago, chamois have been one of the main prey species of the wolf on mountains: the development of some adaptive antipredator strategies should then be expected to prevent local extinctions. In our study area, population dynamics of chamois showed a general decline in growth rate across neighbour valleys irrespectively from wolf recolonisation time, and thus were not attributable to wolf predation. However, after wolf re-establishment in Soana valley, the decline of the kid:female ratio was five times higher, compared to the total population. A contemporary, quick increase in the elevation of female groups, with a decrease in their group size, were found only in this valley, which could suggest an indirect effect of the apex predator on the chamois altitudinal distribution.

Chamois accounted for more than one-third of the wolf diet. In fact, the chamois can be a major prey of the wolf on a local scale [63, 64, 94]. Wild boar populations are expanding across the Alps, and in Italy this ungulate is usually the most used prey over other species [95]. Future work should evaluate whether increasing abundance of alternative prey will lead to a decreased use of a mountain-dwelling ungulate such as the chamois, or whether complex processes will develop, e.g., by increasing predation through ‘apparent competition’ [96].

In simplified predator–prey systems, the return of an apex predator should be expected to impact especially on the most vulnerable prey individuals, i.e., juveniles [9, 97]. Conversely, our study ecosystem included several potential prey species, and the return of a top predator was not followed by a decreased survival of chamois kids. Furthermore, the consistency of growth rate trends across valleys, irrespectively from wolf recolonisation time, suggests that population dynamics was not driven by the wolf. Although predation might have direct effects on prey populations [32], these effects could be weak or virtually absent in anthropogenic landscapes or multi-prey systems [98, 99]. The wolf was not a main driver of moose population trends in both Sweden [100] and Isle Royale [101], although it was shown to reduce survival of wapiti *Cervus canadensis* in Banff National Park [84]. Across Europe, wolf predation has been found to determine effects on abundance of red deer populations only where this canid occurred in sympatry with other large carnivores (brown bear *Ursus arctos* and Eurasian lynx *Lynx lynx*), and the effects of human hunting far exceed the cumulated effect of all three large carnivores together [102]. Furthermore, the wolf and other large carnivores have been shown to affect roe deer density only in relatively poor habitats [103]. Conversely, in the French Alps, recolonising wolves seem to have affected roe deer population growth rate [61]. These findings suggest that, on a broad scale, effects on numbers of different prey species can be context-dependent. In our study, a slight decrease of kid-to-female ratios was observed over the total population. However, upon wolf recolonisation, in Soana valley this decline was five times greater than that in the Orco valley. Wolf predation may have amplified this pre-existing process of juvenile decline, interacting with different drivers as observed elsewhere [104, 105]. Nevertheless, predation on newborn offspring is expected to be limited by the selection of sites close to steep and rocky terrain made by female chamois in the birth season and early weaning periods [106, 107], as well as by the availability of several alternative large prey (especially roe deer and wild boar, [90]). However, the limiting effects of predators on prey populations are not only the result of direct predation, but also of the costs implied in antipredator strategies [36]. Assessing the reasons behind the decline of kids-to-female ratios will therefore require further studies. In fact, the current meteorological change could have altered the pasture quality, which has been reported to affect heavily body mass and winter survival of juvenile mountain ungulates [108–110].

In the Soana valley, after being relatively stable for 12 years, the quick increase in elevation of chamois groups appeared to be in synchrony with the arrival of the wolf. Temperatures increased linearly and gradually throughout our 21 study-years, but elevation only increased after wolf recolonisation. Furthermore, precipitation and zonal NDVI did not show increasing or decreasing trends. Elevations quickly increased, even when accounting for meteorological variables and NDVI. This increase (137 m, Fig. 5) took place in just eight years, representing, to our knowledge, one of the quickest upshifts recorded for this species; e.g. in the Swiss Alps, the elevation of harvested chamois increased by 96 m in 23 years, allegedly due to growing temperatures [111]. Our results suggest that the potential effects of temperature on this elevational trend—if any—may be mediated and accelerated by wolf recolonisation.

At higher elevations, in respect to lower ones, the topography is more heterogeneous and there are more rocky areas, screes and cliffs that could represent important refuge sites for chamois, reducing the perceived predation risk. In a mouflon *Ovis aries* population introduced to an area of the Western Alps, a decrease in the distance from refuge areas was observed especially for females and corresponded to an increase in elevation, slopeness and terrain ruggedness, after wolf recolonisation [112]. Selection for higher elevations was also observed in bighorn sheep, probably because of a combination of foraging requirements and predator avoidance [113]. Conversely, we did not observe the same process in the Orco valley, where the first wolf reproduction occurred in 2017. Antipredator strategies could require some time to develop. The year of first reproduction was used as the start of the period of wolf permanent presence: however, in the Orco valley, solitary wolves were already visiting the valley a few years before, probably increasing the perceived predation risk in chamois even before wolf reproduction occurred. In this area, chamois groups were already located at higher elevations than in Soana valley and may have already met the optimal trade-off between safety and foraging needs.

Temperature was included in a set of candidate models, but they were not selected. Although our results indicate an uplift of chamois after wolf recolonisation, we caution against definite conclusions on the influence of temperature. Interactions should not be ruled out between weather and vegetation (e.g., elevational shifts of preferred food patches, [103]), or by direct effects of temperature on chamois behaviour [114], not captured by our data. If so, the role of the wolf may act as a positive catalyst of an ongoing complex ecological process. For North American herbivores, a remarkable increase in the occupancy of forested habitats has been observed in regions with a warmer climate [115], suggesting that species sufficiently adapted to forests could mitigate the effects of increasing temperature by increasing selection of these habitats. The Northern chamois is a flexible species capable to exploit woodland: in the Eastern Alps, the body mass of harvested chamois living in forest was stable over a 27-year period, while a decreasing trend was detected in animals living in areas with lower forest cover [116]. Recent research on GPS-collared Alpine chamois has reported a preference for increasing tree cover as a response to high summer temperatures [117]. If this species could buffer the negative effects of a warmer temperature by increasing the use of forested habitats, a strong elevational uplift, which increases the distance from the tree line, would be counter-intuitive and may support our conclusion of a predator-driven response.

Simultaneously to the elevational shift, group size significantly decreased in Soana valley after wolf recolonisation. At higher elevations, productivity is lower and resources are scattered, in turn influencing group size [118], although the effects of decreasing chamois density on group size trends cannot be completely ruled out. Although a wealth of studies has suggested that living in large groups, in open landscapes, allows an earlier detection of predators and decreases the probability of being selected as prey [26, 59, 119], one could argue that living in smaller groups might also have an antipredator function as predators should detect larger groups more easily than smaller ones. In wapiti *Cervus canadensis*, large groups suffered more wolf predation and two alternative strategies with comparable predation risk were adopted: living in larger groups benefitting from the dilution effect or in less detectable smaller groups [25]. In multi-prey systems with shared predators, group size variation with increasing predation risk can follow species-specific patterns, either increasing or decreasing [26]. In a valley next to our study area, a decrease in male group size was observed for the ibex and deemed to be an antipredatory behaviour [60].

Whether the decrease in group size is an antipredator strategy or a consequence of abundance and dispersion of food resources at higher altitudes, the ultimate effects on individual fitness are hardly predictable. When escape terrains include marginal, sub-optimal habitats, food acquisition could be limited in quantity or quality [120, 121]. Benefits of group living may be reduced by higher costs in terms of intraspecific competition, parasite load and disease transmission, making it difficult to predict the direction of the effect [122]. If so, staying in large groups may not be convenient, with a trade-off between the benefits of using safer, sub-optimal habitats and those of living in larger groups. Smaller groups face a lower intra-specific competition, which could be a key factor in a gregarious ungulate as the chamois. At the same time, at higher altitudes where resources may be scarcer and more patchy, competition should be more intense and lead to increased aggressive behaviour [123, 124] and decreased maternal care [125].

Despite being the main consumed prey in the area, no clear effect of wolf recolonisation was supported on chamois population dynamics. This result suggests that direct effects of predation on prey numbers were unlikely to be major drivers of population variations [126, 127], whereas indirect effects were suggested through increased elevation. As population-level effects of predators are represented by a combination of direct predation and costs of antipredator strategies [36], the consequences of the observed indirect effects on the future trend of the chamois population should be carefully investigated. Therefore, further research is needed to monitor the evolution of these processes after a longer time of wolf stable presence.

## Supplementary Information

Additional file 1.

## Data Availability

The datasets used and/or analysed during the current study are available from the authors on reasonable request.
