# Supplementary material for: Antipredator behaviour as a major determinant of prey altitudinal movements: the wolf and the chamois
Source: Front Zool. 2025 Aug 21;22:22. doi: 10.1186/s12983-025-00559-1 (PMC12369157; doi:10.1186/s12983-025-00559-1)
Supplement: Supplementary file 1 — Additional file 1. [file 12983_2025_559_MOESM1_ESM.docx]

**Supporting Information**

**S1. Link to Modis terra satellite data:** <https://www.earthdata.nasa.gov/learn/find-data/near-real-time/modis#ed-modis-c61>

**S2: Correlation matrix for the set of covariates:**

**
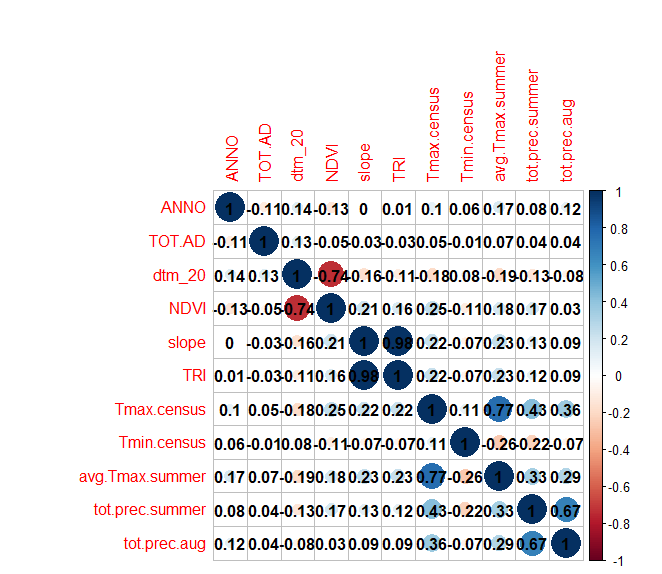
**

**S3: Zonal NDVI trend for each 200m elevation zone.**

**
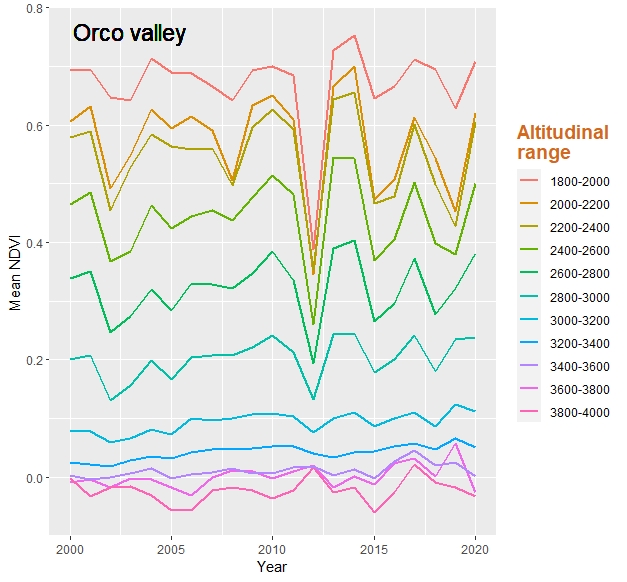
**


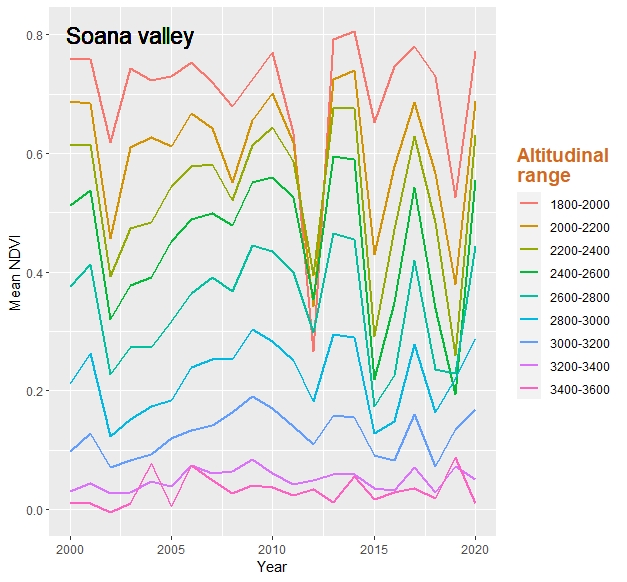


**S4 Complete model selection table for variables explaining chamois elevation and group size. Selected models are shown in italic bold.**

| Variable | Rank | Model structure | df | logLik | AIC | ΔAIC | Weight |
| --- | --- | --- | --- | --- | --- | --- | --- |
| *Elevation* | ***1st*** | ***Wolf + Valley + Wolf * Valley + slope + Tmax survey*** | ***8*** | ***-25674*** | ***51364.6*** | ***0.00*** | ***0.281*** |
|  | ***2nd*** | ***Wolf + Valley + Wolf*Valley + slope*** | ***7*** | ***-25675*** | ***51365.0*** | ***0.42*** | ***0.231*** |
|  | 3rd | Wolf + Valley + Wolf*Valley + avg. summer Tmax + Tmax survey + slope | 9 | -25673 | 51365.6 | 1.04 | 0.17 |
|  | 4th | Wolf + Valley + Wolf*Valley + avg. summer Tmax + slope | 8 | -25675 | 51366.4 | 1.81 | 0.115 |
|  | 5th | Wolf + Valley + Wolf*Valley + Tmax survey + tot. prec. august + slope | 9 | -25674 | 51366.4 | 1.81 | 0.115 |
|  | 6th | Wolf + Valley + Wolf*Valley + tot. prec. august + slope | 8 | -25675 | 51367.0 | 2.39 | 0.086 |
| *Group size* | ***1st*** | ***Wolf + Valley + Wolf*Valley + NDVI*** | ***7*** | ***-10982.87*** | ***21979.8*** | ***0.00*** | ***0.127*** |
|  | 2nd | Wolf + Valley + Wolf*Valley + NDVI + tot. prec. august | 8 | -10982.03 | 21980.1 | 0.33 | 0.106 |
|  | 3rd | Wolf + Valley + Wolf*Valley + NDVI + Tmax survey | 8 | -10982.03 | 21980.1 | 0.34 | 0.106 |
|  | 4th | Wolf + Valley + Wolf*Valley + NDVI + Tmax survey + tot.prec.august | 9 | -10981.13 | 21980.3 | 0.54 | 0.096 |
|  | 5th | Wolf + Valley + Wolf*Valley + NDVI + avg. summer Tmax | 8 | -10981.35 | 21980.7 | 0.97 | 0.077 |
|  | 6th | Wolf + Valley + Wolf*Valley + NDVI + avg. summer Tmax + Tmax survey | 9 | -10981.36 | 21980.8 | 1.00 | 0.076 |
|  | 7th | Wolf + Valley + Wolf*Valley + NDVI + avg. summer Tmax + Tmax survey + tot.prec.august | 10 | -10980.44 | 21980.9 | 1.18 | 0.069 |
|  | 8th | Wolf + Valley + Wolf*Valley + NDVI + slope | 8 | -10982.48 | 21981.0 | 1.24 | 0.067 |
|  | 9th | Wolf + Valley + Wolf*Valley + NDVI + tot.prec.august + avg. summer Tmax | 9 | -10981.51 | 21981.1 | 1.31 | 0.065 |
|  | 10th | Wolf + Valley + Wolf*Valley + NDVI + slope + Tmax survey | 9 | -10981.64 | 21981.3 | 1.57 | 0.057 |
|  | 11th | Wolf + Valley + Wolf*Valley + NDVI + slope + tot.prec.august | 9 | -10981.67 | 21981.4 | 1.63 | 0.055 |
|  | 12th | Wolf + Valley + Wolf*Valley + NDVI + slope + tot.prec.august + Tmax survey | 10 | -10980.76 | 21981.6 | 1.82 | 0.050 |
|  | 13th | Wolf + Valley + Wolf*Valley + NDVI + slope + avg. summer Tmax | 9 | -10981.96 | 21982.0 | 2.20 | 0.043 |
